# Analgesic Efficacy of Spinal Morphine in Comparison With Transversus Abdominis Plane Block for Postoperative Pain Management in Patients Undergoing Cesarean Section Under Spinal Anesthesia: A Randomized Controlled Trial

**DOI:** 10.3389/fmed.2022.814538

**Published:** 2022-02-09

**Authors:** Bedru Jemal, Fetiha Mohammed, Hailemariam Getachew Tesema, Siraj Ahmed, Ayub Mohammed, Teshome Regasa, Mohammed Suleiman Obsa

**Affiliations:** ^1^Department of Anesthesiology, College of Health Sciences and Medicine, Dilla University, Dilla, Ethiopia; ^2^Department of Anesthesiology, College of Health Sciences and Medicine, Hawasa University, Dilla, Ethiopia; ^3^Department of Anesthesia, College of Medicine and Health Science, Wollo University, Dessie, Ethiopia; ^4^Department of Anesthesiology, College of Health Sciences and Medicine, Woliata Sodo University, Dilla, Ethiopia

**Keywords:** spinal morphine, TAP, analgesia, cesarean section, randomized controlled trial, postoperative pain management

## Abstract

**Background:**

Cesarean section (CS) has been one of the most frequently performed major surgical interventions and causes severe postoperative pain. Spinal opioid and abdominal field block have been investigated as effective analgesia for postoperative pain and reduce the need for systemic medications and associated side effects. The aim of the current study is to compare spinal morphine (SM) and bilateral landmark oriented transversus abdominis plane (TAP) block for postoperative pain management.

**Method:**

In this randomized controlled trial, 114 pregnant mothers scheduled for CS under spinal anesthesia were allocated randomly to receive either SM 0.1 mg (group SM; *n* = 56) or bilateral landmark-oriented TAP block with 20 ml of 0.25% of bupivacaine (group TAP; *n* = 52). A comparison of numerical variables between study groups was done using unpaired student *t*-test and Mann–Whitney test for symmetric and asymmetric data, respectively. Time to event variable was analyzed by using Kaplan–Meir's survival function. A *p*-value of < 0.05 was considered statistically significant.

**Result:**

A total of 114 patients were recruited and randomly assigned and received interventions. Among them, 108 patients completed this study. Time to first analgesic request was significantly shorter in the TAP block compared to SM. Twenty-four-hour median morphine consumption was reduced in the SM group compared to the TAP block (*p* < 0.001). Median postoperative pain score during movement and rest shows statistically significant differences between groups (*p* < 0.001).

**Conclusion:**

The addition of preservative-free 100 μg SM provides prolonged postoperative analgesia time, superior postoperative analgesia, and less postoperative opioid consumption compared to the TAP block.

## Introduction

Cesarean section (CS) is a commonly performed major surgical procedure that results in substantial postoperative pain and patient dissatisfaction (1–3). The rates of CS in developing countries continue to rise as a result of changing patterns in obstetrics practice and maternal requests (4). The skin, abdominal wall, and uterine incisions for cesarean delivery generate postoperative pain. Different nerve fiber subgroups convey skin and visceral nociception, and the pattern of discharge is controversial. The uterine afferent fibers stimulated primarily include C-fibers with some A-delta fibers. However, the majority of afferent fibers that relay nociceptive stimuli from the skin are A-delta fibers. This particular component brings about the difference in pain sensation and requires different types of pain management protocols (5, 6). As a result of the involvement of two different types of pain receptors, there is difficulty in managing pain after cesarean delivery (6).

Management of postoperative pain is challenging, with 30–80% of women experiencing moderate-to-severe pain (7). Approximately 79% of the women who undergo CS experience pain at the incision site that lasts for up to 2 months. It is associated with moderate-to-severe postoperative pain and is reported that the intensity of the pain is equivalent to that of hysterectomy (4). Adequate pain management after CS benefits not only the parturient and the baby but also the healthcare system. Inadequately managed pain can lead to difficulty in mobility, increased risk of venous thrombosis, interferences in optimal interaction with the newborn, predispositions to splinting, and risk of chest infections (8, 9). Hence, the provision of effective postoperative analgesia is important in facilitating the early mobilization of a parturient, provision of optimal infant care, prevention of postoperative morbidity, enhancement of patient satisfaction, shortening the duration of hospital stay (7, 10, 11), and reducing the extra burden on healthcare (12).

Although different options for pain management exist, the ideal form of postoperative analgesia is still unknown. The clinical efficacy of transverse abdominis plane (TAP) block and SM has been demonstrated in different clinical trials of patients undergoing abdominal surgery including CS showed different effects regarding postoperative analgesic consumption and pain score (13, 14). In addition, the comparison of landmark-oriented TAP block vs. SM has not been well addressed in the literature. So conducting this study in a setting where there is a lack of resources is very important to improve the quality of postoperative pain and patient satisfaction.

## Methods and Materials

### Study Design, Setting, and Sample Population

A prospective, parallel-randomized, controlled, investigator-blinded, single-center trial was conducted from January 2020 to July 2020. Study participants were included using a systematic random sampling method from consecutive patients. All parturients (aged between 18 and 45 years) who underwent elective cesarean delivery *via* Pfannenstiel incision under spinal anesthesia were included. Patients who refused to participate, ASA 3 and 4 patients, failure to administer spinal anesthesia, patients with a history of chronic pain disorder, patients who were allergic to local anesthetics and morphine, patients with cognitive impairment, and patients with preeclampsia were excluded from this trial.

### During the Preoperative Period

Preanesthetic evaluation was done in the morning on the day of surgery, and eligibility criteria were checked before recruiting the study participants. Informed consent was obtained from each participant. Vital signs, organ function tests, history, and physical examinations were reviewed. On arrival to the operating room, parturients were premedicated with IV cimetidine (200 mg), metoclopramide (10 mg), dexamethasone (4 mg), and ondansetron 0.1 mg/kg 30 min before the induction of anesthesia for prevention of aspiration, nausea, vomiting, and pruritus according to the institutional protocol. Non-participating researchers prepare morphine *via* double dilution procedures. It was started by drawing 1 ml of 10 mg/ml morphine in a 10-ml syringe, and this was diluted with 9 ml of 0.9% normal saline. After mixing, again 1 ml was drawn from a syringe and diluted with 9 ml of 0.9% normal saline. Finally, after mixing, 1 ml (100 mcg) of the mixture was aspirated and added with bupivacaine.

### Intraoperative Period

Standard monitoring non-invasive blood pressure (NIBP), oxygen saturation (SPO_2_), heart rate, heart rhythm, end-tidal carbon dioxide, and temperatures were monitored every 5 min and recorded every 10 min. Spinal anesthesia was then administered between L3 and L4 with 12.5 mg of 0.5% heavy bupivacaine with 100 μg preservative-free morphine by using a 24-gauge spinal needle for spinal morphine (SM) group and spinal anesthesia with 12.5 mg of 0.5% heavy bupivacaine and bilateral landmark-oriented TAP block with 20 ml, 0.25% bupivacaine at each side for the TAP group at the end of the operation. The blocks were performed by an anesthetist with >2 years of experience in the performance of TAP block.

### Postoperative Period

During follow-up visits in the postoperative period, patient's blood pressure, heart rate, and pain scores were assessed using a numerical rating scale (NRS) and managed according to the protocol. After the parturient was discharged from operation theater, pain severity was assessed by NRS at 2nd, 6th, 12th, and 24th h at rest and movement. Those patients with NRS score beyond 3 points were managed with tramadol 1 mg/kg IV, diclofenac 1 mg/kg IV, and paracetamol 20 mg/kg PO. Patients with NRS scores beyond 7 points were managed with diclofenac 1 mg/kg IV, paracetamol 20 mg/kg PO, and morphine 0.1 mg/kg IV as per the protocol. Tramadol that was used during the postoperative period was converted to morphine equivalent dose using a 0.1 conversion ratio (15). Following surgery, postoperative adverse events, such as nausea, vomiting, pruritus, and respiratory depression, were recorded and managed according to the protocol. The severity of postoperative nausea and vomiting (PONV) was determined according to the verbal numerical rating scale (VNRS) score: mild (1–3), moderate (4–6), and severe (7–10). Pruritus intensity was measured using a 5-point verbal rating scale (VRS): no pruritus (0 points), mild pruritus (1 point), moderate pruritus (2 points), severe pruritus (3 points), and very severe pruritus (4 points). Respiratory depression was monitored using pulse-oximetry and respiratory rate. Respiratory depression is defined as one episode of respiratory frequency of < 10 breaths/minute within the first 24 h or oxygen saturation < 90%. Following surgery, the patient was monitored continuously for 2 h in the post-anesthesia care unit, allowing for continuous registration of oxygen saturation, heart rate, and non-invasive BP every 10 min. After the first 2 h, clinical assessments for respiratory depression were conducted every 4 h for the first 6 h, then every 6 h for the next 6 h, and finally at 12-h intervals.

### Randomization and Blinding

Patients were allocated using computer-generated randomization. During allocation, the researcher did not participate in either SM or TAP block. To achieve blinding to the intervention, a drape was used to prevent women from visualizing the performance of the TAP block. Various data collectors were employed during the intraoperative and postoperative periods. The intraoperative caregiver was not blinded to the intervention group.

### Study Outcomes Measures and Endpoints

The primary outcome was the time to first analgesic request. Time to first analgesic request was recorded upon patient request. Secondary outcomes were postoperative pain intensity, analgesic consumption, the proportion of patients who achieved adequate analgesia, adverse effects (pruritus, nausea, vomiting, and sedation), and maternal satisfaction with the quality of analgesia. Postoperative pain intensity was measured by a standardized NRS at 2, 6, 12, and 24 h postoperatively.

### Sample Size and Statistical Analysis

To show a clinically significant 50% difference in time to first analgesic request between SM and TAP block groups, with 80% statistical power and a two-tailed significance level of 5%, each group required 57 subjects and 114 subjects in total.

Data were checked, coded, entered, and analyzed by the IBM SPSS version 25 software package (IBM Corp., Armonk, NY, USA). The data were tested for normality by using the Shapiro–Wilk test and homogeneity of variance by Levene's test for continuous data. Numerical data were described in terms of mean ± SD for symmetric and median (interquartile range, IQR) for asymmetric data. A comparison of sociodemographic variables, hemodynamic data, intraoperative tramadol consumption, duration of surgery, and Apgar score between study groups was performed by using unpaired Student's *t*-test, and Mann–Whitney test was used for pain intensity and morphine equivalent analgesic consumption. Frequency and percentages were used to describe categorical variables. Statistical difference between groups was tested by Chi-square and Fisher exact test according to the nature of data. Time to event variable was analyzed using Kaplan–Meir's survival function. Generalized Estimation Equation (GEE) were used to model the repeatedly assessed NRS pain scores longitudinally to investigate the interactive effects of the intervention group and repeated time. Autoregressive Order 1 (AR1) structure of working correlation matrix was assumed and SEs were used. The independent variables were the follow-up time and intervention group. The effects and interactions between follow-up time to the intervention were assessed. A *p*-value of < 0.05 was considered statistically significant.

### Ethical Clearance Approval

The study received approval from the Dilla University Institutional Review Board (Dilla, Ethiopia; protocol: 004/19-10; December 20/2019). In addition, this study has been prospectively registered in December 2019 at Pan African Clinical Trial Registry with the identification number PACTR202002616299138. The purpose, importance, and risk of the study were explained to each participant prior to obtaining written informed consent. Participants were informed that they could withdraw from the study without any restrictions at any time.

## Result

**Figure 1:**
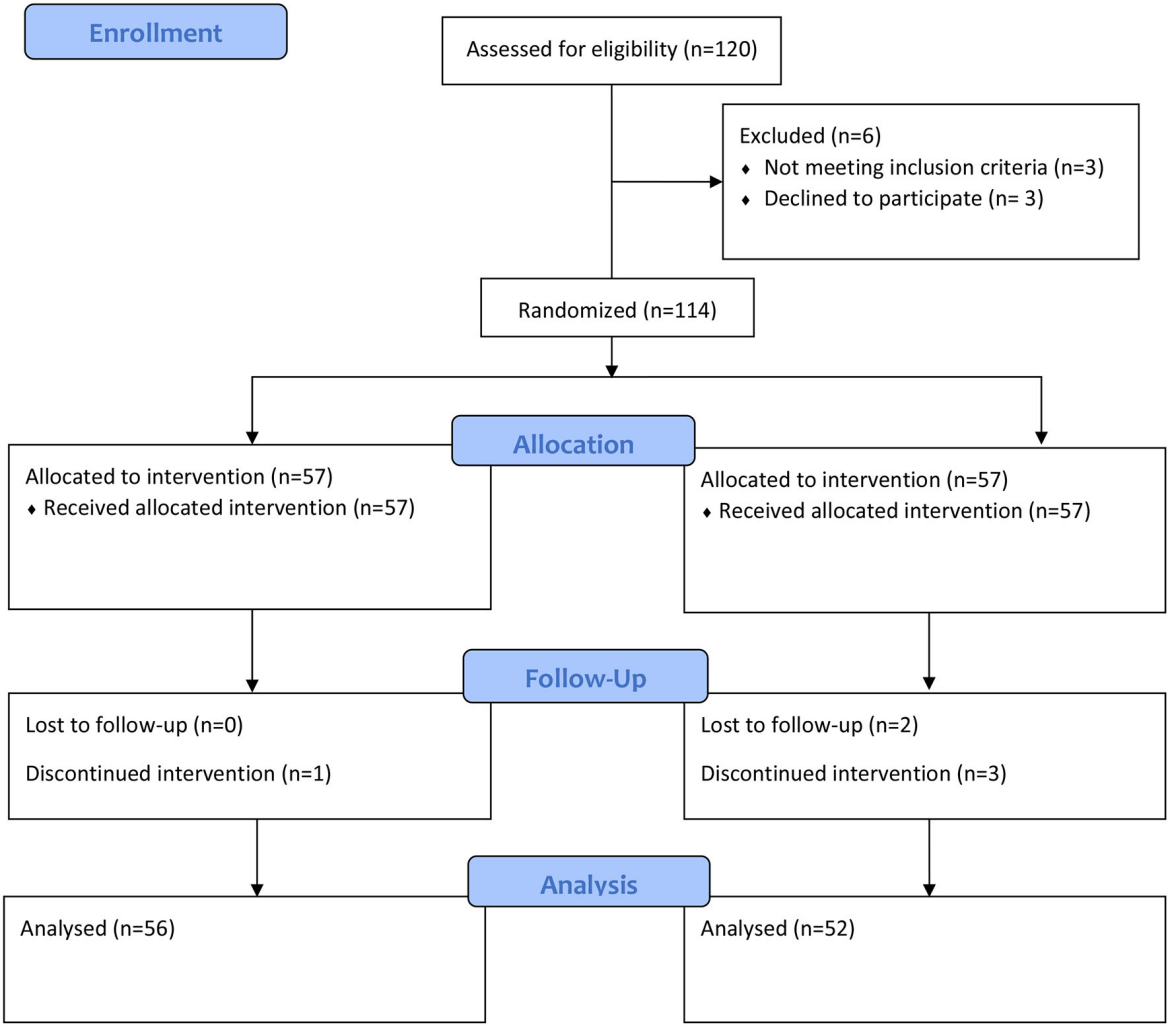
Consort flow diagram of patient enrolment.

Out of 114 patients who were screened for this prospective, randomized, controlled study, 108 patients were finally recruited and randomly assigned to the groups (Figure 1). Among all patients, three of them were excluded due to refusal for participation and three due to failure to meet inclusion criteria. After allocation, four patients were changed to general anesthesia, whereas two cases were discharged from the hospital during follow-up. In total, 108 patients completed this study. Except for height and gestational age, no significant variations in age, body mass index, operation length, or anesthetic were found between groups when sociodemographics and baseline clinical characteristics were compared (Table 1).

**Table 1:** Sociodemographic characteristics of patients who underwent elective cesarean section (CS) under spinal anesthesia.

**Variable**	**SM**	**TAP**
Age (years)	27.39 ± 4.65	28.1 ± 5.52
Height (m)	1.64 ± 0.04	1.66 ± 0.06
Weight (kg)	71.05 ± 9.11	72.6 ± 9.35
Body mass index	25.3 ± 2.68	25.46 ± 2.68
Gestational age (weeks)	38.7 ± 0.62	38.5 ± 0.5
Baseline heart rate	91.17 ± 14.6	89.65 ± 14.32
Baseline diastolic blood pressure (mmHg)	77.6 ± 9.55	76.48 ± 1.98
Baseline systolic blood pressure (mmHg)	129.78 ± 16	127.65 ± 11.3
Time taken to administer spinal anesthesia (s)	6.76 ± 1.9	7.3 ± 2.1
Parity		
Multipara	45 (80.4)	46 (88.56)
Ethnicity		
Sidama	20 (35.7)	22 (42.3)
Oromo	33 (58.9)	23 (44.2)
Others	3 (5.4)	7 (13.5)

*Data are mean ± SD or number (%). TAP, transversus abdominis plane; SM, spinal morphine*.

There was no statistically significant difference observed regarding the level of motor block, sensory block, and duration of surgery between the groups with a *p*-value of > 0.05 (Table 2).

**Table 2:** Comparison of perioperative factors in elective caesarian delivery under spinal anesthesia and randomized to either SM or a TAP block for postoperative pain management.

**Variable**	**SM**	**TAP**	***p*** **-value**
Level of sensory			0.278
T4	54 (96.4)	47 (90.46)	
T5	0 (0.0)	2 (3.8)	
T6	2 (3.6)	3 (4.6)	
Level of motor			0.229
Grade (4)	0 (0)	2 (1.9)	
Grade (5)	56 (100)	50 (96.2)	
Nausea			0.83
Yes	14 (25)	14 (26.9)	
Vomiting			0.115
Yes	3 (5.4)	8 (15.4)	
Apgar	8.7 ± 0.62	8.4 ± 0.67	0.064
Intraoperative tramadol rescue dose (mg)	36.6 ± 11.54	20 ± 0.0	0.148
Intraoperative rescue tramadol	3 (5)	2 (4)	0.708
Duration of surgery	48.3 ± 4.79	47.8 ± 5.17	0.663
Duration of anesthesia	122.14 ± 5.94	123.6 ± 13.25	0.441

*T4&T5, thoracic vertebra 4&5; SM, spinal morphine; TAP, transversus abdominis plane. Data are mean ± SD or number (%)*.

### Time to First Analgesic Request

**Figure 2:**
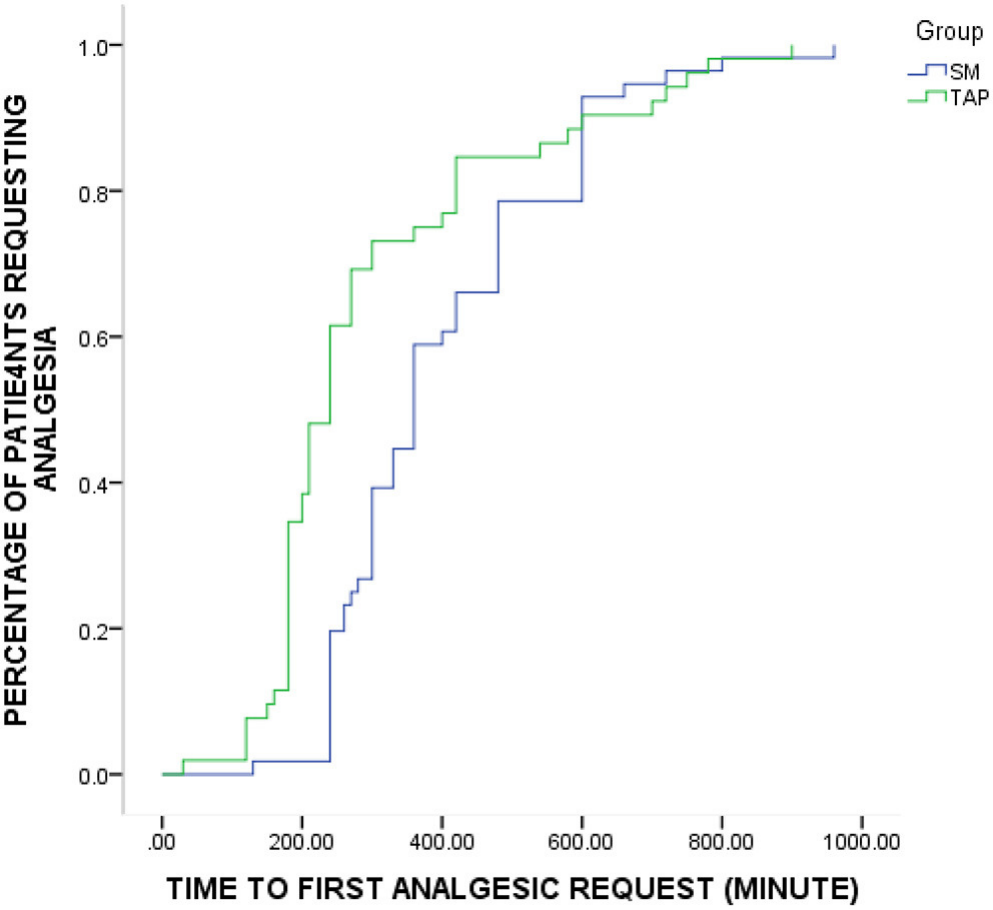
Kaplan–Meier curve depicting the proportion of patients in each group over time who require supplemental analgesic (*p* = 0.004, log-rank test). SM, spinal morphine; TAP, transversus abdominis plane.

Time to first analgesic request was significantly shorter in the TAP group compared to the SM group. Particularly, the patients in the SM group, median time: 360 min, 95% CI: [332–387] had significantly longer time to first analgesic request compared to the TAP group, median time: 240 min 95% CI: [217–262] (*p* = 0.004) (Figure 2). The cumulative proportion of patients requesting analgesia at the time 4 h after surgery in the SM group was 20% compared to 62% in the TAP group.

### Postoperative Pain Intensity

**Figure 3:**
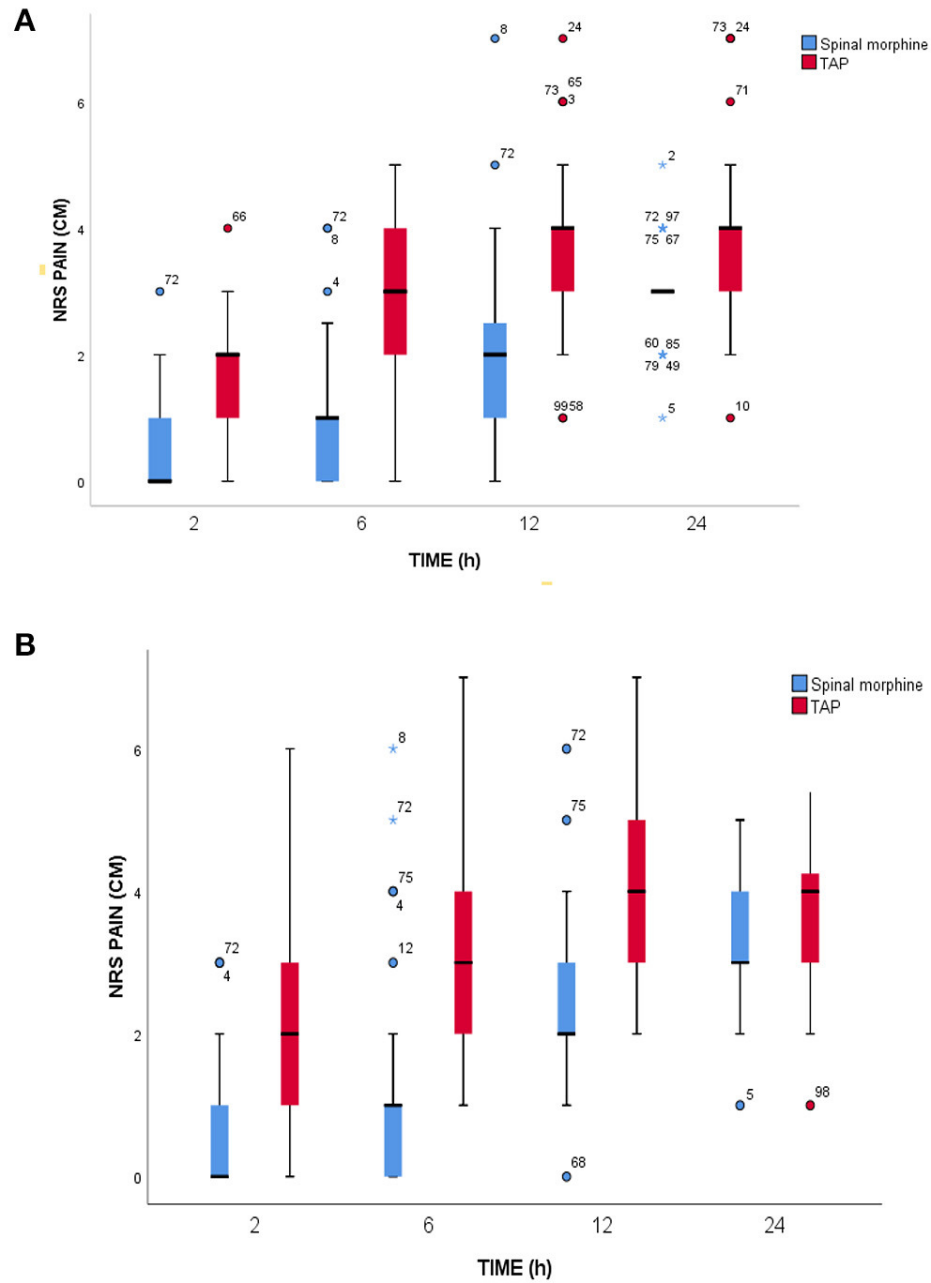
Postoperative pain scores. **(A)** At rest. **(B)** During movement at a different time point during the 24-h assessment period. SM, spinal morphine; TAP block, transversus abdominis plane block; NRS, numerical rating score; cm, centimeter; IQR, interquartile range. The horizontal line indicates median, and hinges indicate first and third quartiles. Whiskers extend to the largest and smallest values not more than 1.5 × IQR. Outlier data points beyond the end of the whiskers are plotted as individual dots, and numbers in outlier data represent the serial number of individual data.

Postoperative pain intensity scores at different points of time between SM and TAP block were significantly different, as assessed by numerical pain rating scale. The reported median pain score at rest during the first 2 h was 0 (0–1) in the SM group and 2 (1–2) in the TAP group (*p* < 0.001). Similarly, in the 6, 12, and 24 h postoperatively, median (IQR) NRS score was 1 (0–1) vs. 3 (2–4) (*p* < 0.001), 2 (1–3) vs. 4 (3–4) (*p* < 0.001), and 3 (3–3) vs. 4 (3–4) (*p* < 0.001) in the SM and TAP groups, respectively. Median postoperative pain scores during movement at 2nd, 6th, 12th, and 24th h were 0.5 (0–1) vs. 2 (1–3) (*p* < 0.001), 1 (0–1) vs. 3 (2–4) (*p* < 0.001), 2 (2–3) vs. 4 (3–5) (*p* < 0.001), and 3 (3–4) vs. 4 (3–5) (*p* < 0.001) in the SM and TAP groups, respectively (Figures 3A,B).

### Group and Repeated Measure Interaction

After adjustment of pain score at rest for repeated measurements, generalized estimating equation model showed that NRS score was 0.9 units lower in the SM group than in the TAP group during the first 24-h postoperative period in NRS pain scores at rest (β = −0.9, 95% CI = −1.3 to −0.5, Wald's χ^2^ = 20.8, *p* < 0.001). Generalized Estimation Equation (GEE) indicated that there was a statistically significant interaction between the intervention group and repeated measure at 6th and 12th h (β = −0.827, 95% CI = −1.3 to −0.33, Wald's χ^2^ = 10.9, *p* = 0.001 and β = −0.769, 95% CI = −1.2 to −0.31, Wald's χ^2^ = 11, *p* = 0.001 respectively).

### Repeated Measure

There were statistically significant effects of repeated measurement in pain score at rest 2nd h (β = −2.4, 95% CI = −2.78 to−2.01, Wald's χ^2^ = 168, *p* < 0.001 6th h postoperatively (β = −1.3, 95% CI = −1.7, to −0.9, Wald's χ^2^ = 41.7, *p* < 0.001) in the GEE model.

### Intervention Group and Repeated Measurement Interaction

**Figure 4:**
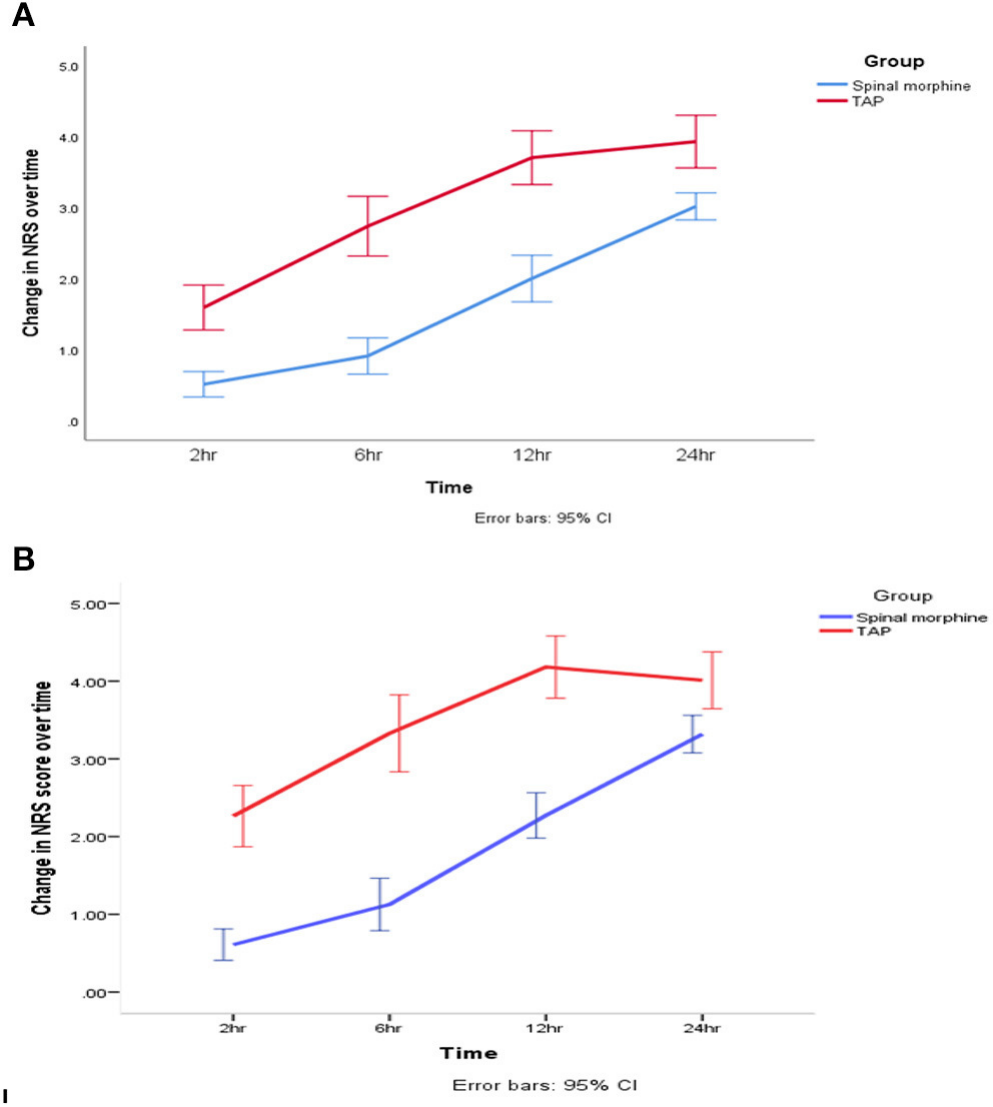
Change in postoperative pain score. **(A)** At rest. **(B)** On movement during the 24-h postoperative period. SM, spinal morphine; TAP block, transversus abdominis plane block; NRS, numerical rating score. The line indicates mean, and hinges indicate 95% CI.

After adjustment of pain score during movement for repeated measurement, generalized estimating equation model shows NRS score was 0.74 unit lower in the SM group than in the TAP group during the first 24-h postoperative period (β = −0.74, 95% CI = −1.13 to −0.34, Wald's χ^2^ = 13.2, *p* < 0.001) (Figures 4A,B).

The GEE model showed that there was a statistically significant interaction between the intervention group and repeated measurement during movement pain score at 2nd h (β = −0.88, 95% CI = −1.3 to −0.42, Wald's χ^2^ = 14.06, *p* < 0.001), 6th h (β = −1.42, 95% CI = −1.9 to −0.9, Wald's χ^2^ = 33.00, *p* < 0.001), and 12th h (β = −1.19, 95% CI = −1.63 to −0.75, Wald's χ^2^ = 28.08, *p* < 0.001).

### Repeated Measurement

There were statistically significant effects of repeated measurement on pain intensity score (NRS) during movement at 2nd h (β = −1.8, 95% CI = −2.2 to −1.4, Wald's χ^2^ = 79.10, *p* < 0.001) and 6th h postoperatively (β = −0.75, 95% CI = −1.14 to −0.35, Wald's χ^2^ = 13.8, *p* < 0.001).

### Postoperative Analgesic Consumption

**Figure 5:**
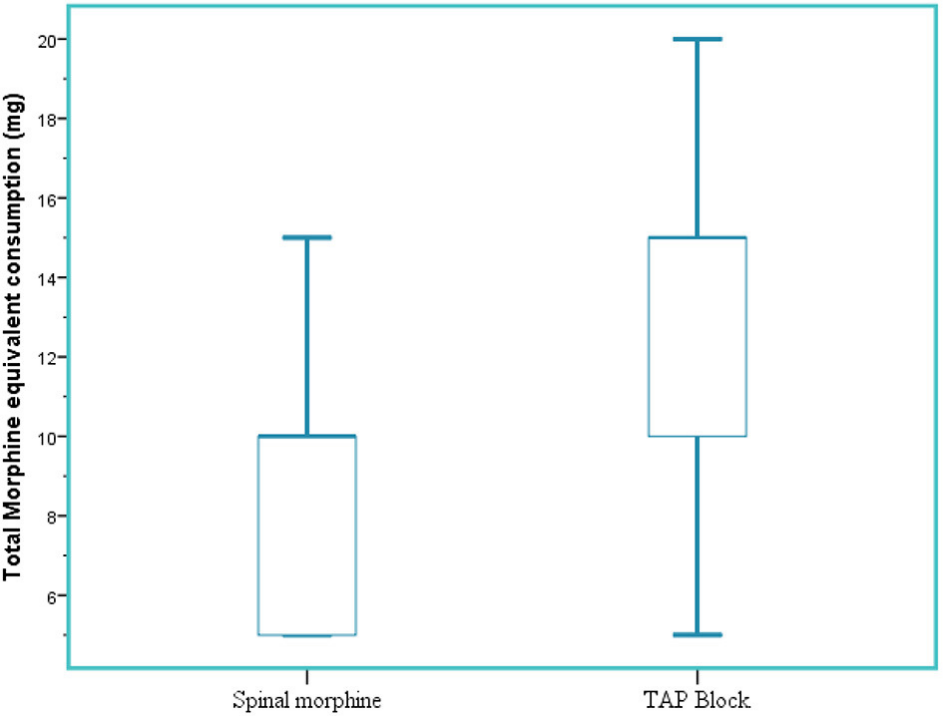
Total 24-h morphine equivalent analgesic consumption. SM, spinal morphine; TAP block, transversus abdominis plane block; mg, milligram; IQR, interquartile range. The horizontal line indicates median, and hinges indicate first and third quartiles. Whiskers extend to the largest and smallest values not more than 1.5 × IQR.

In the immediate postoperative period (2 h), median tramadol consumption was 0 ± (0) mg in the SM group and 12.5 ± (21.8) mg in the TAP group (*p* < 0.001. Twenty-four-hour median morphine equivalent consumption was reduced in the SM group 5 (5–10) mg compared to the TAP block group 10 (10–15) mg (*p* < 0.001) (Figure 5).

### Adequacy of Analgesia

At rest, 62.6% of patients in the SM group reported pain scores ≤3 compared with 37.4% in the TAP group. The SM group was significantly associated with adequate analgesia at rest (pain scores ≤3) compared to the TAP group (OR: 1.96, 95% CI: 1.34–2.57, 0.001). On movement, 65.8% of patients in the SM group had pain scores ≤3 as opposed to 34.2% in the TAP group. The SM group was significantly associated with adequate analgesia during movement (pain scores ≤ 3) compared to the TAP group (OR: 1.70, 95% CI: 1.14–2.25, *p* < 0.001).

### Postoperative Complications and Patient Satisfaction

None of the patients in the study developed respiratory depression. Two patients from the SM group were lightly sedated, and one patient had moderate pruritus but did not require treatment. The majority of the parturients rated that their satisfaction was higher with SM than that of the TAP group. There was a statistically significant difference regarding postoperative satisfaction between the two groups. There were no statically significant differences in sedation, nausea, and pruritus scores between the two groups (Table 3).

**Table 3:** Postoperative complication and satisfaction of parturients underwent elective cesarean section under spinal anesthesia and randomized to receive spinal morphine or TAP block for postoperative pain management.

**Variable**	**SM**	**TAP**	***p*-value**
Ramsay sedation score			0.49
Awake	54 (96.4)	52 (100)	
Slightly sedated	2 (100)	0	
Postoperative nausea			0.496
No	46 (82.1%)	46 (88.5%)	
Mild	10 (17.9%)	4 (7.7%)	
Moderate	0 (0)	1 (1.9%)	
Severe	0 (0)	1 (1.9%)	
Pruritus			1.00
None	55 (98.2%)	52 (100%)	
Moderate	1 (1.8%)	0 (0)	
Satisfaction			<0.001
Highly satisfied*	45 (19.6%)	14 (23.74)	
Satisfied	11 (19.6%)	36 (69.2%)	
Dissatisfied	0 (0)	1 (1.9%)	
Respiratory depression	0 (0)	0 (0)	

## Discussion

The post-CS pain delays the healing process and prolongs the recovery of the patient. Acute pain after delivery is a cause of depression, and it is difficult to deliver postoperative pain, which is suitable for both patients and children (15, 16). This study found that SM provided better analgesic efficacy with prolonged time to first analgesic request, lower pain score, and less opioid consumption compared to that of the TAP group. Time to first analgesic request was significantly longer in the SM group with a median time of 360 min compared to 240 min in the TAP group, which is relatively shorter compared with 12 h described in the literature (17). Similarly, another randomized controlled trial showed that intrathecal morphine (ITM) provides better analgesia than that of the TAP block (18). In addition, ITM is associated with prolonged median time (480 min) of supplemental analgesia request, lower VAS pain scores, and less tramadol consumption after CS. The findings in the study explained the effectiveness of ITM to treat somatic and visceral pain arising from the wound and the uterus, respectively (18). Contrary to our study, previous studies did not find a significant difference between the groups (19, 20). This difference might be explained by the technique of the TAP block they used and the use of adjuvant opioids in addition to SM, which might affect the duration and intensity of analgesia.

The results of our study showed that SM exhibited significantly lower pain scores at rest and on movement during the postoperative period at 2, 6, 12, and 24 h compared with the TAP group. The results of our study also showed that SM greatly improved 24-h postoperative pain intensity at rest after cesarean deliveries compared to the TAP group. On the other hand, another similar study found that median VRS scores were 10 mm and 26.5 mm at rest during the first 24 h (21). The reason for the difference in pain intensity score between these studies might be explained by the difference in the tool used for pain score assessment, medications, and the volume of local anesthetics used for both TAP and spinal anesthesia.

This study found a significant difference in analgesic consumptions between groups. There was less need for analgesia in the SM group than in the TAP group. Twenty-four-hour median morphine equivalent analgesic consumption is significantly reduced in the SM group (median consumption 5 mg higher in the TAP group), as a result of effective analgesia in the postoperative period. Similar to our findings, an earlier study found that the TAP block was associated with greater supplemental morphine equivalent requirements of 7.5 mg (95% CI 4.8–10.2) than the SM 2.7 mg (95% CI 1.0–4.3) (22).

Regarding postoperative complications (sedation, nausea, vomiting, and pruritus), results between the two groups did not show any clinically significant difference (17). This can be explained by the relatively low-dose SM we used (100 mcg), compared to the commonly used dose of 200 mcg. Furthermore, the routine use of multimodal analgesia protocol using paracetamol and diclofenac may lead to opioid to sparing effect, which leads to less postoperative complications like postoperative nausea, vomiting, pruritus, and respiratory depression.

In our study, all patients received 4 mg of ondansetron before the procedure for prophylaxis of vomiting and pruritus. Postoperative maternal satisfaction with the quality of pain relief was significantly different between the two intervention groups. Most of the patients in the SM group were highly satisfied with the quality of pain control compared to that of the TAP group. In contrary to our study, previous studies have shown maternal satisfaction was similar for both SM and TAP groups (18–20). The difference was explained by the content assessed for maternal satisfaction. In the earlier study, there was an assessment of overall maternal satisfaction; however, in the present study, maternal satisfaction regarding the quality of pain control and the satisfactory levels of the patient with the pain management was thoroughly assessed. Respiratory depression has not been observed after intrathecal administration of morphine at doses of 0.1 mg for CS and was not seen in our study either (19, 20).

### Limitations and Strengths of the Study

This is a single-center study. Although the study population is inclusive, the external generalizability of these results has taken into account the size of the study population. However, the results of the study are presented consistently. In this study, ultrasound was not used for the TAP block because of the lack of ultrasound for nerve block in the study area. Organ penetration and other rare complications associated with the TAP block were not examined. We did not assess the incidence of postdural puncture headache (PDPH); however, we used the same gauge needles for both groups to ensure that they were equally affected. Another limitation of our study was that we did not assess the success rate or sensory spread of the resulting block, because a residual sensory block from spinal anesthetic might last for hours after surgery. Additionally, there were difficulties in adequately blinding intraoperative caregivers and data collectors. However, neither the patients nor postoperative data collectors were aware of group allocations. Another limitation of this study was we did not assess the comparison of SM (not difficult, reliable technique), with a blind TAP block (a technique known to be more reliable with the guidance of ultrasound and that only might control the somatic pain) is *a priori* expected to be more favorable for the SM group. Therefore, we recommend future researches to assess PDPH and explore other comparisons like SM with the TAP block of other dosages.

## Conclusion and Recommendations

We conclude that the addition of preservative-free 100 μg SM for cesarean delivery under spinal anesthesia provides superior analgesia, prolonged time to first analgesic request, and less postoperative opioid consumption compared to that of the landmark TAP block. No significant differences in postoperative complications like nausea, vomiting, and pruritus between groups were found. Thus, we recommend the use of preservative-free 100 μg SM for parturients who undergo CS under spinal anesthesia to achieve prolonged time to first analgesic request, for better postoperative analgesia, and to reduce the side effects of analgesic consumption.

## Clinical Trial Registration

This study is registered with the Pan African Clinical Trial Registry on 24 December 2019 and the registration number was PACTR202002616299138 (https://apps.who.int/trialsearch/Trial2.aspx?TrialID$=$PACTR202002616299138).

## Data Availability Statement

The original contributions presented in the study are included in the article/Supplementary Materials, further inquiries can be directed to the corresponding author/s.

## Ethics Statement

The studies involving human participants were reviewed and approved by Dilla University Institutional Review Board. The patients/participants provided their written informed consent to participate in this study.

## Author Contributions

BJ and FM have made substantial contributions in conception, design, analysis, interpretation of data, critical review, and editing of the manuscript drafts for scientific merit and depth. SA, AM, TR, HT, and MO have made substantial intellectual contributions to the acquisition of data, interpretation of data, and preparing the manuscript for this study. All authors have read and approved the final version of the manuscript.

## Conflict of Interest

The authors declare that the research was conducted in the absence of any commercial or financial relationships that could be construed as a potential conflict of interest.

## Publisher's Note

All claims expressed in this article are solely those of the authors and do not necessarily represent those of their affiliated organizations, or those of the publisher, the editors and the reviewers. Any product that may be evaluated in this article, or claim that may be made by its manufacturer, is not guaranteed or endorsed by the publisher.
